# Regulation of hPCL3 isoforms’ ubiquitination by TRIM21 in non-small cell lung cancer progression

**DOI:** 10.26508/lsa.202302060

**Published:** 2023-07-28

**Authors:** Ye Xu, Wenhong Liu, Xiawei Jiang, Junfeng Li, Qingling Liu, Fang Su, Shanming Ruan, Zhiqian Zhang, Fangfang Tao

**Affiliations:** 1 Department of Immunology and Microbiology, School of Basic Medical Sciences, Zhejiang Chinese Medical University , Hangzhou, China; 2 Department of Medical Oncology, First Affiliated Hospital of Zhejiang Chinese Medical University , Hangzhou, China; 3 State Key Laboratory of Medicinal Chemical Biology, Nankai University, Tianjin, China

## Abstract

Non-small cell lung cancer (NSCLC) is the main subtype of lung cancer. The role of hPCL3 isoforms, hPCL3S and hPCL3L, remains ambiguous. This study examines the functional implications of these isoforms in NSCLC, using lung cancer cell lines A549 and NCI-H226c for in vivo and in vitro analyses. The results indicate that elevated expression of both hPCL3S and hPCL3L correlates with diminished overall survival, although only hPCL3S levels are augmented in clinical NSCLC specimens. Inhibition of either isoform leads to reduced cell proliferation, invasion, and migration, with hPCL3S knockdown displaying superior effectiveness. Moreover, the findings reveal that TRIM21 interacts with both isoforms and mediates hPCL3S degradation through K48-linked ubiquitination in NSCLC cells. Conversely, TRIM21 does not facilitate hPCL3L degradation, despite forming K63-linked polyubiquitin chains. These observations highlight the divergent roles of hPCL3 isoforms in NSCLC and underscore the potential therapeutic value of targeting hPCL3S.

## Abstract (precis)

hPCL3 isoforms, hPCL3S and hPCL3L, are studied in NSCLC. Elevated expression of both isoforms correlates with poor survival. Inhibiting hPCL3S reduces cell growth, invasion, and migration, suggesting it as a potential therapeutic target.

## Introduction

According to World Cancer Report 2020 produced by the International Agency for Research on Cancer, lung cancer was the second most diagnosed cancer (11.4%) and the leading cause of cancer-related deaths (18.0%) in 2020 (Sung et al, 2021). In 2021, an estimated 235,760 American adults (119,100 men and 116,660 women) will be diagnosed with lung cancer and 131,880 lung cancer–related deaths are estimated to occur. In China, there were ∼820,000 new patients occurred in 2020, and 710,000 people died of lung cancer, accounting for 23.8% of all cancer deaths (International Agency for Research on Cancer, 2018). Non-small cell lung cancer (NSCLC) is the most predominant subtype of lung cancer, ∼85% of all lung cancer cases were identified as NSCLC (Thomas et al, 2015; Rotow & Bivona, 2017). NSCLC includes squamous cell carcinoma, adenocarcinoma, and large cell carcinoma. Currently, gene alterations have been found in two-thirds of clinical specimens from patients with NSCLC (Rotow & Bivona, 2017). Thus, to better understand the progression and metastasis of NSCLC, and to propose therapeutic targets and prognostic indicators for clinical diagnosis and treatment, it is crucial to explore the molecular mechanism of NSCLC.

The polycomb repressive complex 2 (PRC2) plays pivotal roles in development, differentiation, and cell fate determination by catalyzing the methylation of histone H3 lysine 27 (H3K27) (Jones & Wang, 2010; He et al, 2012; Liu et al, 2018). Mutation and deregulation of the PRC2-encoding genes often promote cancer (Comet et al, 2016; Wassef & Margueron, 2017). The polycomb-like protein 3 (PCL3), also called PHD finger protein 19 (PHF19), has been proposed to modulate the enzymatic activity of PRC2 which was first identified in *Drosophila* (Duncan, 1982). PCL3 achieves this regulation primarily through its direct interaction with PRC2. This interaction is facilitated by the TUDOR domain present in PCL3, which recognizes and binds to the trimethylated lysine residues on histone H3, leading to recruitment and stabilization of PRC2 on chromatin. Consequently, PRC2 is enabled to catalyze the methylation of H3K27, contributing to transcriptional repression (Ballaré et al, 2012; Brien et al, 2012). Human PCL3 (*hPCL3*) gene was first identified in 2004 and its products are notably highly expressed in various human tumor types and are explicitly related to aggressive tumor behavior (Boulay et al, 2012; Cai et al, 2018; Abdelfettah et al, 2020). There are two isoforms of hPCL3 that have been reported: a shorter isoform (hPCL3S, 207 aa) and a long isoform (hPCL3L, 580 aa) (Wang et al, 2004). hPCL3S contains only the N-terminal TUDOR domain and the first PHD1 domain. Both hPCL3S and hPCL3L interact with the enhancer of zeste homolog 2 (Boulay et al, 2011). Moreover, hPCL3S and hPCL3L exhibit strikingly distinct subcellular localization patterns (Boulay et al, 2011), suggesting that hPLC3S has different functions from hPCL3L.

In this study, we examined the expression level of hPCL3S and hPCL3L in clinical NSCLC samples. We demonstrated that both hPCL3S and hPCL3L regulate NSCLC cell migration and invasion in vivo and in vitro. The interaction proteins of hPCL3S and hPCL3L have been screened using FLAG affinity purification and mass spectrometry assays. We also found that TRIM21 could interact with both hPCL3S and hPCL3L and promotes K48-linked ubiquitination of hPCL3S and K63-linked ubiquitination of hPCL3L. This study explored the diverse roles of hPCL3S and hPCL3L in promoting the proliferation and migration of NSCLC.

## Results

### Compared with hPCL3L, hPCL3S was significantly up-regulated in NSCLC and associated with lower survival in NSCLC patients

**Figure 1.**
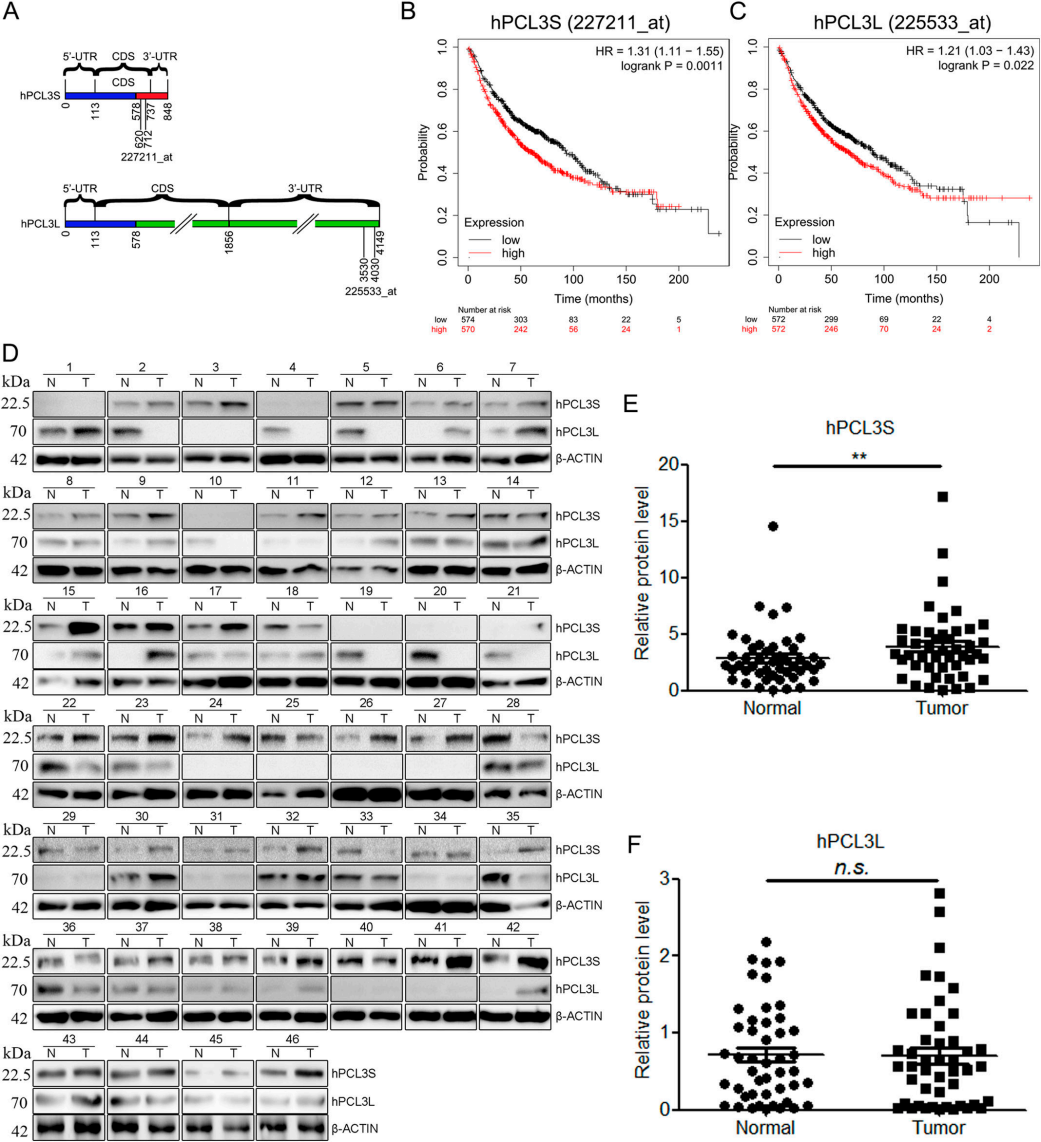
hPCL3S was up-regulated in NSCLC and predicts a poorer prognosis for NSCLC patients compared with hPCL3L. **(A)** Schematic diagram showing the transcripts of *hPCL3S* and *hPCL3L*. Same sequence shared by both isoforms is marked by the blue box. Affymetrix GeneChip probe 227221_at uniquely targets the 620–712 nt of *hPCL3S* CDS. Affymetrix GeneChip probe 225233_at uniquely targets the 3,530–4,030 nt of *hPCL3L* CDS. **(B, C)** Kaplan–Meier analyses for overall survival of 1,925 patients with different levels of hPCL3S (B) and hPCL3L (C). Survival curves were constructed using the Kaplan–Meier method and were analyzed by the log-rank test. **(D)** The expression levels of hPCL3S and hPCL3L in tumor tissues of 46 NSCLC patients compared with corresponding healthy controls (para-carcinoma tissues). Western blotting was performed to detect the protein expression of hPCL3S and hPCL3L. **(E)** The relative protein levels of hPCL3S were quantified. **(F)** The relative protein levels of hPCL3L were quantified. Data are presented as means ± SEM. **, *P* < 0.01. *n.s.*, non-significant.

There are two isoforms of *hPCL3*, *hPCL3S* (848 nt) and *hPCL3L* (4,149 nt), which share the same sequence as the first 578 nt (Fig 1A). The critical efficiency of *hPCL3S* or *hPCL3L* in survival of patients with NSCLC was explored using Kaplan–Meier plotter (www.kmplot.com) (Győrffy, 2021). Specific Affymetrix GeneChip probe was selected as shown in Fig 1A to distinguish the expression level of *hPCL3S* and *hPCL3L*. Kaplan–Meier survival analyses were performed in 1925 NSCLC patients. The results indicated that higher *hPCL3S* or *hPCL3L* mRNA expression in patients with NSCLC was significantly correlated to lower over survival, in which *hPCL3S* (HR 1.13 [1.11–1.55], *P* = 0.0011) (Fig 1B) showed a more significant correlation than that of *hPCL3L* (HR 1.21 [1.03–1.43], *P* = 0.022) (Fig 1C).

The expression of hPCL3S and hPCL3L in tumor and normal tissue of 46 patients with NSCLC were quantified by Western blotting (Fig 1D). The results demonstrated that the expression of hPCL3S is significantly increased in the tumor of patients with NSCLC compared with that of normal tissues (Fig 1E), whereas the expression of hPCL3L was not significantly changed (Fig 1F). These results suggested hPCL3S may play a more powerful role in promoting NSCLC progression compared with hPCL3L.

### Knockout of hPCL3S shows more powerful effect on suppressing NSCLC cell growth and mobility in vitro compared with that of hPCL3L

**Figure S1.**
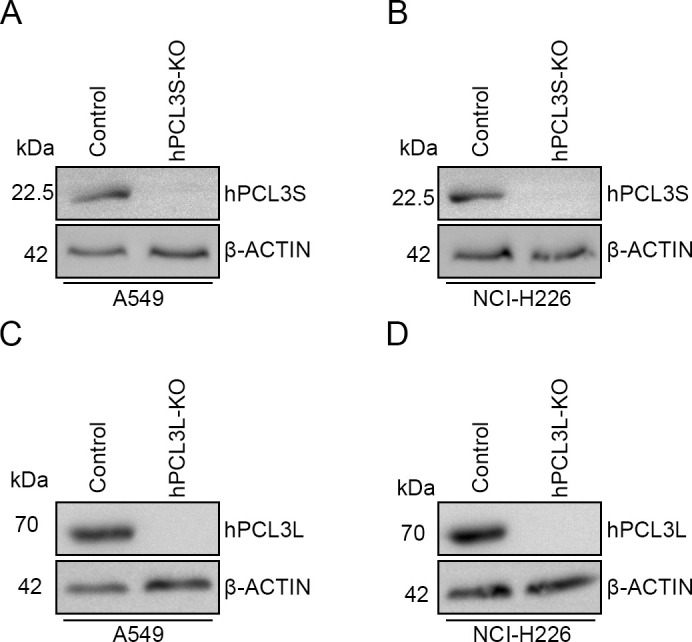
Knockout of hPCL3S and hPCL3L in A549 cells and NCI-H226 cells. **(A)** hPCL3S knockout in A549 cells. **(B)** hPCL3S knockout in NCI-H226 cells. **(C)** hPCL3L knockout in A549 cells. **(D)** hPCL3L knockout in NCI-H226 cells.

**Figure S2.**
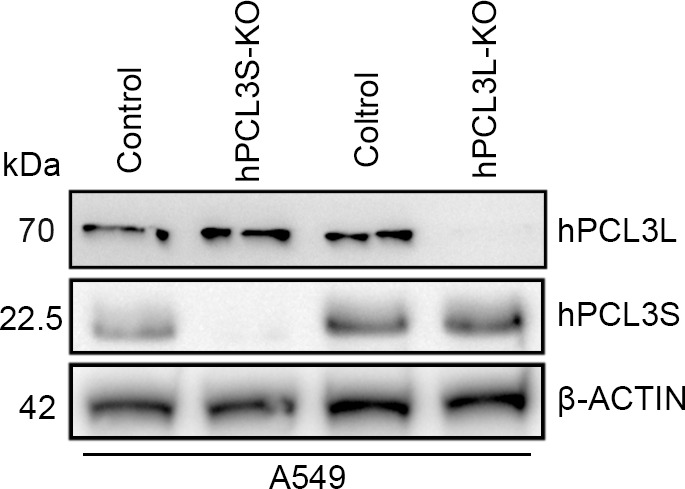
Knockout of hPCL3S and hPCL3L in A549 cell.

**Figure 2.**
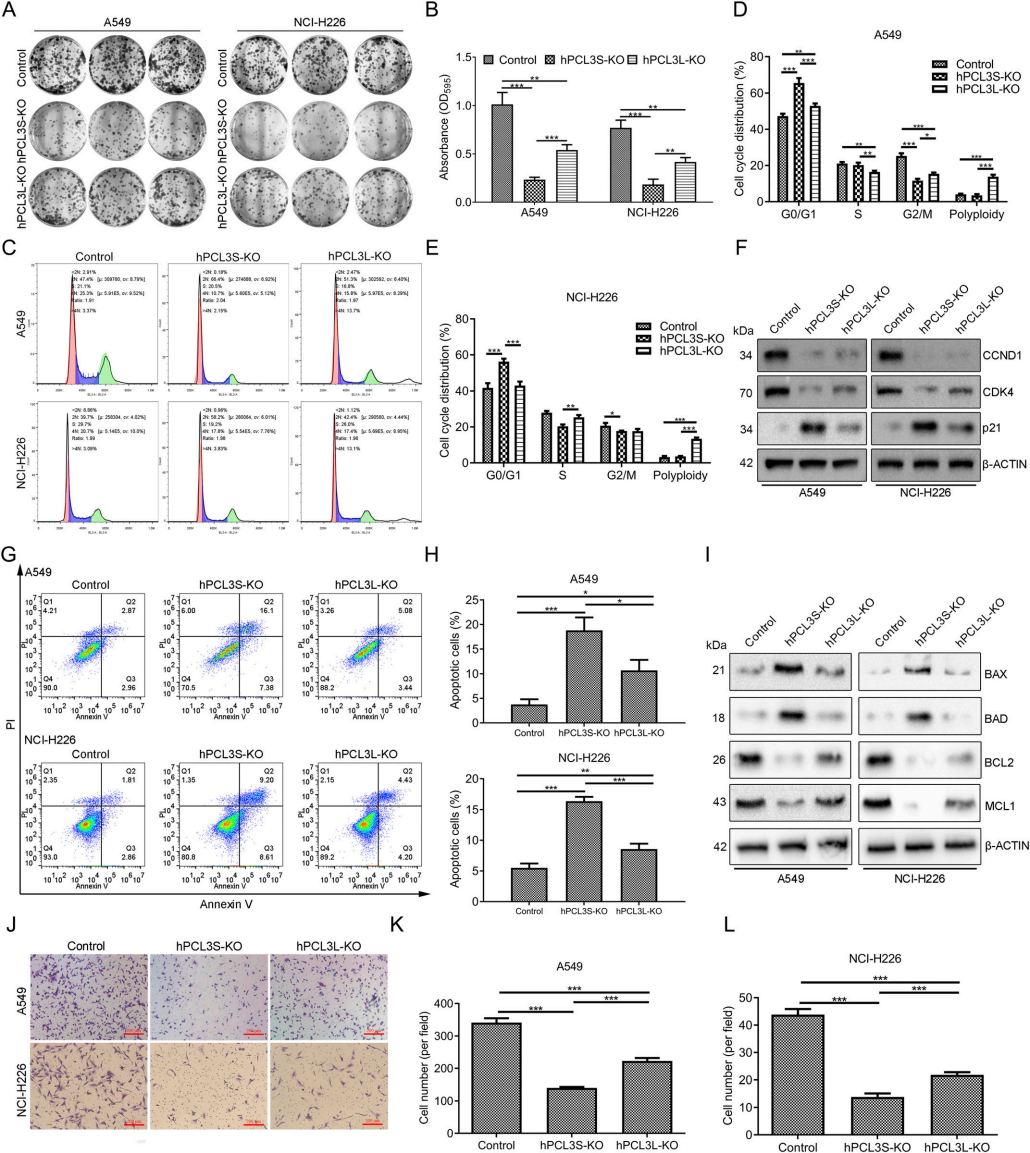
Effect of hPCL3S and hPCL3L knockout on proliferation, cell cycle, apoptosis, and migration of A549 and NCI-H226 cells. **(A)** Colony formation assay to evaluate the effect of hPCL3S or hPCL3L knockout on A549 (left) and NCI-H226 (right) cell proliferation. **(A, B)** Quantification of colony formation in (A). Optical density of solubilized and eluted crystal violet from stained colonies. **(C)** Flow cytometry to determine the cell cycle distribution in A549 cells and NCI-H226 cells with hPCL3S or hPCL3L knockout. **(C, D, E)** Quantification of cell cycle distribution in (C). **(F)** The expression profiles of the cell cycle–related proteins in hPCL3S-KO, hPCL3L-KO, and control cells. **(G)** The apoptosis rate of A549 and NCI-H226 cells with hPCL3S or hPCL3L knockout. **(G, H)** Quantification of cell apoptosis in (G). **(I)** The expression profiles of apoptosis-related proteins in hPCL3S-KO, hPCL3L-KO, and control cells. **(J)** Cell invasion ability in hPCL3S-KO, hPCL3L-KO, and control cells were examined using transwell chamber, and invaded cells were stained with crystal violet and counted (**P < 0.01; ***P < 0.001). **(J, K, L)** Quantification of cell number in (J). Data shown are mean values of three independent experiments performed in duplicate.

Next, we intended to investigate the effect of hPCL3S and hPCL3L on NSCLC progression. Therefore, hPCL3S (Fig S1A and B) or hPCL3L (Fig S1C and D) was knocked out in NSCLC A549 and NCI-H226 cells using the CRISPR/Cas9 system. Knockout efficiencies were confirmed by Western blotting (Fig S2). First, the colony formation assays were performed by growing hPCL3S-KO, hPCL3L-KO, and control cells for 14 d. We found both the colony formation capacity of hPCL3S-KO and hPCL3L-KO cells were severely inhibited compared with control cells (Fig 2A and B). Moreover, the hPCL3S-KO cells reflected lower colony formation rate than hPCL3L-KO cells.

Subsequently, we examined the effects of hPCL3S and hPCL3L on cell cycle progression in A549 cells and NCI-H226 cells by using flow cytometry assay. Cell cycle analysis revealed that hPCL3S-KO increased the proportion of cells in the G0/G1 phase of A549 cells (from 48% to 70%) and NCI-H226 cells (from 40% to 58%) and accompanied with a reduction in the proportion of A549 cells (from 25% to 15%) and NCI-H226 cells (from 22% to 18%) in G2/M phase. Knockout of hPCL3L also led to a moderate increase in the proportion of A549 cells in G0/G1 phase (from 48% to 52%) and accompanied with a reduction in the proportion of A549 cells in the S phase (from 20% to 15%) and G2/M phase (from 25% to 17%) (Fig 2C–E). The Western blotting results showed that both hPCL3S and hPCL3L knockout induced p21 expression and inhibited cyclin D1 (CCND1) and CDK4 in A549 and NCI-H226 cell lines; the change rate in hPCL3S cells was higher than that in hPCL3L cells (Fig 2F). These data demonstrate that knockout of both hPCL3S and hPCL3SL inhibits cell proliferation possibly through arresting cell cycle at G0/G1 phase, whereas hPCL3S knockout has a stronger effect on inhibiting cell proliferation in NSCLC. In addition, results of apoptosis assays indicated that both hPCL3S and hPCL3L knockout significantly increased the percentage of apoptotic cancer cells compared with the control A549 and NCI-H266 cells (Fig 2G and H). Moreover, the hPCL3S-KO cells showed a higher efficiency of inducing apoptosis than that of the hPCL3L cells. The Western blotting results showed that hPCL3S-KO–induced BAX and BAD expression and inhibited BCL2 and MCL1 in A549 and NCI-H226 cells (Fig 2I). Furthermore, hPCL3L-KO showed less effect in regulating the expression changes of these proteins. In the transwell assay, the invasiveness of A549 and NCI-H226 cells both in the hPCL3S-KO and hPCL3L-KO group was significantly reduced compared with control cells. Moreover, the invasiveness of the hPCL3S-KO group was significantly lower than that of the hPCL3L-KO group in both A549 and NCI-H226 cells (Fig 2J–L). Overall, these results indicated that both hPCL3S and hPCL3L knockout inhibited NSCLC cells proliferation, invasion, and induced its apoptosis and migration. Our results also implied the suppression effect of hPCL3S showed a deeper impact than that of hPCL3L.

### Knockout of hPCL3S exhibits a higher inhibitory effect on tumor growth and metastasis in NSCLC cells compares with hPCL3L knockout

**Figure 3.**
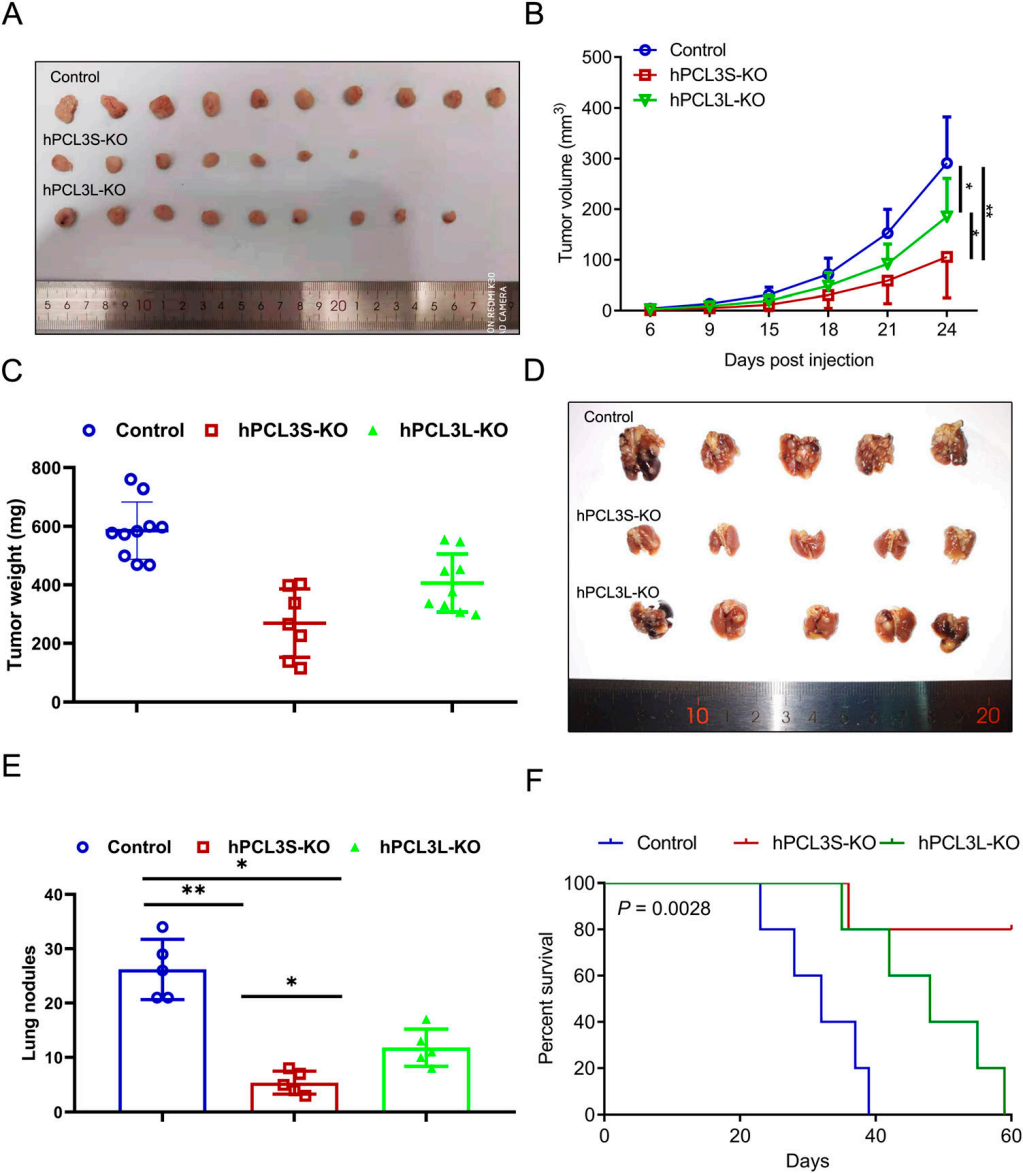
Effect of hPCL3S and hPCL3SL knockout in tumor growth and metastasis of NSCLC cells in vivo. **(A)** Representative pictures of tumors from A549 xenografts in the groups of control, hPCL3S-KO, or hPCL3S-KO, respectively. **(B)** The tumor volume in mice was measured every 3 d from the 6th d. The statistical significance of the difference between treatments were assessed using *t* test (**P < 0.01). **(C)** Tumor weight of control, hPCL3S-KO or hPCL3S-KO group. **(D)** The images of lung metastases. Control, hPCL3S-KO, or hPCL3S-KO A549 cells were injected intravenously by tail vein into Balb/c nude mice. Representative pictures of fixed lungs at 6 wk after injection are shown. **(D, E)** Quantification of metastasis nodules in the control and hPCL3S-KO or hPCL3S-KO tumors from (D). **(F)** In vivo survival assay of mice after control and hPCL3S-KO or hPCL3SL-KO cells were injected intravenously by tail vein. The significance was measured by *t* test. NS, not significant; *P < 0.05; **P < 0.01; ***P < 0.001.

Furthermore, we confirmed the pro-tumorigenic role of hPCL3S and hPCL3L in vivo. We tested the effect of hPCL3S-KO and hPCL3L-KO cells on tumor growth in Balb/c nude mice. Compared with the control group, both the hPCL3S-KO and hPCL3L-KO groups suppressed tumor growth in mice, resulting in significant reductions in tumor size (Fig 3A), volume (Fig 3B), and weight (Fig 3C). Also, the tumor size, volume, and weight of the hPCL3S-KO group were significantly smaller or less than that of the hPCL3L-KO group. To establish a metastasis model, control, hPCL3S-KO, or hPCL3L-KO cells were injected into the tail vein of Balb/c nude mice. Mice were euthanized, and lung metastases were counted 42 d post-injection. The number of lung metastases was significantly decreased in both hPCL3S-KO and hPCL3L-KO groups compared with the control group, whereas the hPCL3S-KO group had less metastases than the hPCL3L-KO group (Fig 3D and E). The Kaplan–Meier survival assay showed that the cumulative survival rate and median survival time in hPCL3S-KO mice significantly decreased compared with that in hPCL3L-KO and control mice (log-rank tests, *P* < 0.05) (Fig 3F). These results demonstrate that hPCL3S and hPCL3L played an intrinsic role in promoting tumor formation and progression. Besides, hPCL3S showed a significantly more powerful effect than hPCL3L.

### TRIM21 interacts with hPCL3S and hPCL3L

**Figure 4.**
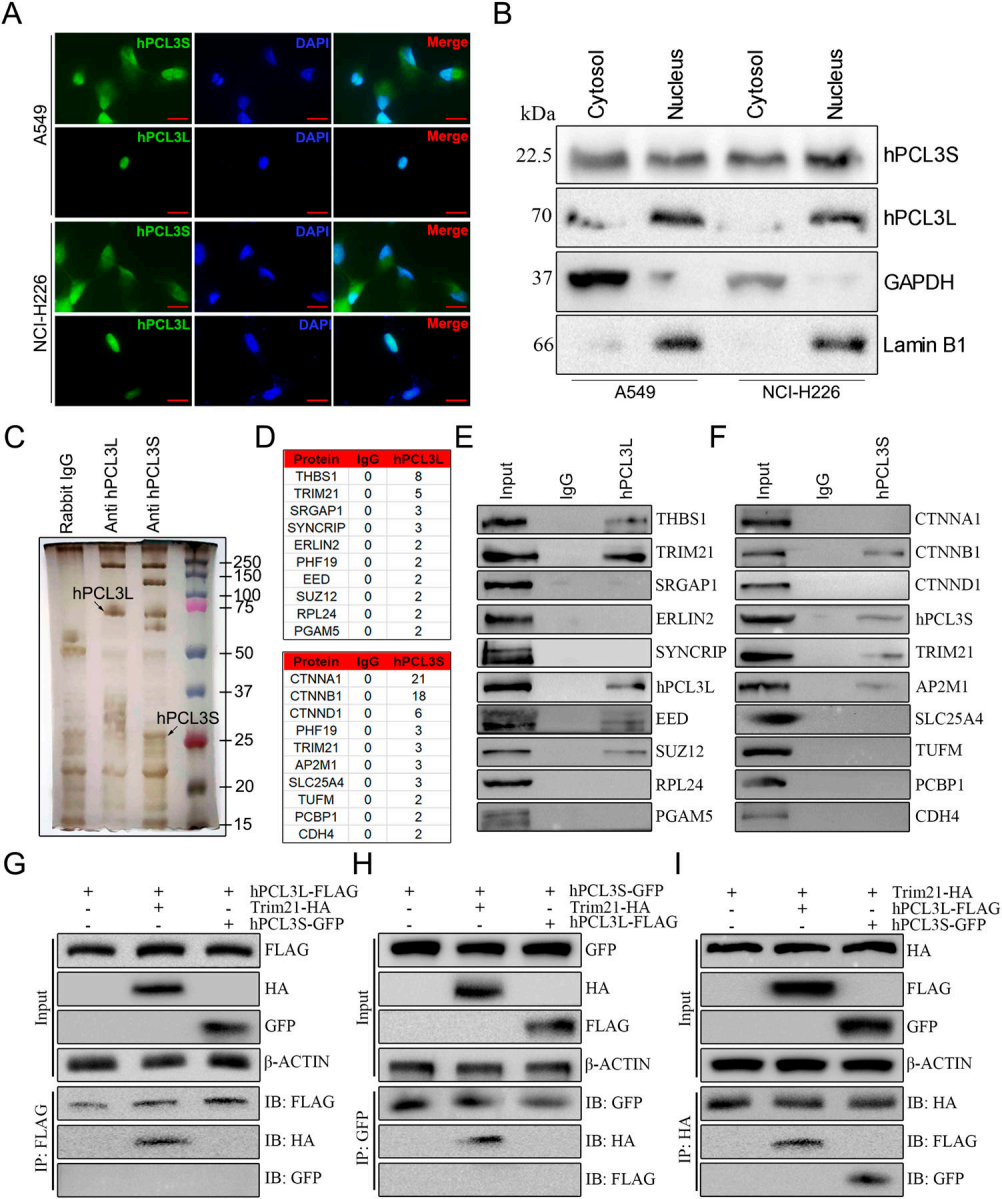
Subcellular localization of hPCL3S and hPCL3SL in NSCLC cells and TRIM21 interacted with both hPCL3S and hPCL3L. **(A)** Subcellular localization of hPCL3S and hPCL3L in A549 cells and NCI-H226 cells. **(B)** Cytosolic and nuclear extracts were applied for Western blotting detection of hPCL3S, hPCL3SL in A549 cells and NCI-H226 cells. GAPDH and Lamin B1 were used as controls of the quality of the cytoplasmic and nuclear protein fractions, respectively. **(C)** SDS–PAGE and silver staining of the proteins immunoprecipitated by hPCL3S or hPCL3SL antibodies, respectively. Lane 1, the sample immunoprecipitated by Rabbit IgG. Lane 2, the sample immunoprecipitated by anti-hPCL3L antibody in A549 cell lysate. Lane 3, the sample immunoprecipitated by anti-hPCL3S antibody in A549 cell lysate. **(D)** The top ten ranked proteins that were identified in the indicated immunoprecipitants using mass spectrometry. **(E)** Western blotting to confirm the interaction of the top ten ranked proteins with hPCL3S, respectively. **(F)** Western blotting to confirm the interaction of the top ten ranked proteins with hPCL3S, respectively. **(G, H, I)** The interaction among hPCL3S, hPCL3L, and TRIM21. **(G)** hPCL3S directly interacts with TRIM21 in vivo. **(H)** hPCL3L directly interacts with TRIM21 in vitro. **(I)** Trim21 directly interacts with hPCL3S or hPCL3L.

To further explore the subcellular localization and expression of hPCL3S and hPCL3L in NSCLC cells, immunofluorescence assays and Western blotting analysis were performed in A549 cells and NCI-H226 cells. As showed in Fig 4A, hPCL3S was detected in both cytosol and nucleus. But hPCL3L was only detected in the nucleus of both A549 cells and NCI-H226 cells. The Western blotting assay validated this conclusion (Fig 4B). To gain insight into the interacting proteins of hPCL3S and hPCL3L, SDS–PAGE and silver staining were performed after immunoprecipitation (IP) of hPCL3S and hPCL3L (Fig 4C). The top ten ranked proteins of hPCL3S and hPCL3L were identified by mass spectrometry, respectively (Fig 4D). In addition, Western blotting of the IP product confirmed that TRIM21 was one of the proteins that interact with both hPCL3S and hPCL3L (Fig 4E and F). Furthermore, co-immunoprecipitation (Co-IP) experiments in 293T cells revealed that TRIM21 bound to hPCL3L and hPCL3S in 293T cells (Fig 4I). However, FLAG-tag hPCL3L did not interact with GFP-tag hPCL3S (Fig 4G and H). Taken together, these findings suggested that TRIM21 could directly interact with both hPCL3S and hPCL3L.

### TRIM21 promotes the degradation of hPCL3S but not hPCL3L in NSCLC cells

**Figure 5.**
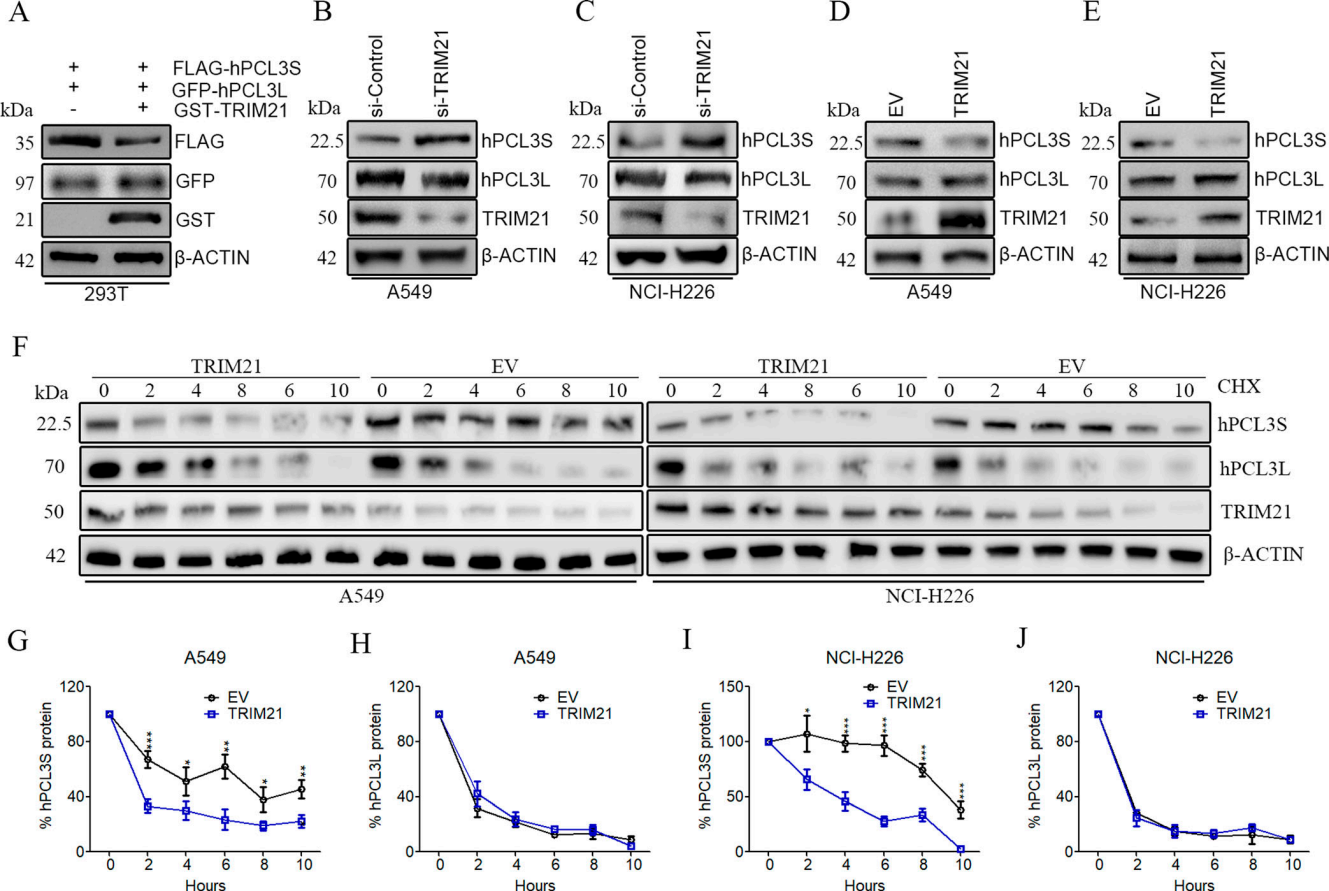
TRIM21 regulates the expression level of hPCL3S. **(A)** Overexpression of TRIM21 only decreased the protein level of hPCL3S in 293T cells. **(B, C)** Knockdown TRIM21 only up-regulated the protein level of hPCL3S both in A549 cells and NCI-H226 cells. **(D, E)** Overexpression of TRIM21 only decreased the protein level of hPCL3S in 293T cells both in A549 cells and NCI-H226 cells. **(F)** Cycloheximide chase assay in empty vector and TRIM21-overexpressed cells. Western blot analysis was performed for hPCL3S, hPCL3L, and β-ACTIN (loading control). Cells were treated with 10 μg/mL cycloheximide to block protein synthesis over a 10-h time period. Data represent one of the three independent experiments with similar results. **(F, G, H, I, J)** Quantitative analysis of the Western blotting results shown in (F) using ImageJ software: (G) hPCL3S in A549, (H) hPCL3L of A549, (I) hPCL3S of NCI-H226, (J) and hPCL3L of NCI-H226. Data represent means ± SD of three assays.

Previous studies illustrated that tripartite motif containing-21 (TRIM21) is a cytosolic ubiquitin ligase (Frank et al, 1993; Reymond et al, 2001; Ozato et al, 2008; Strandberg et al, 2008). Protein ubiquitination leads to several posttranslational effects (Bednash & Mallampalli, 2016; Tsuchida et al, 2017). Thus, the effects of TRIM21 on hPCL3S and hPCL3L were explored. FLAG-hPCL3S and GFP-hPCL3L were overexpressed in 293T cells. After overexpressed GST-TRIM21, only FLAG-hPCL3S expression level was reduced (Fig 5A). Moreover, the expression levels of hPCL3S and hPCL3L were detected in TRIM21-KD and TRIM21-overexpressed A549 cells and NCI-H226 cells using Western blotting. The results showed that hPCL3S protein expression level was elevated in TRIM21-KD A549 (Fig 5B) and NCI-H226 cells (Fig 5C) and was reduced in TRIM21-overexpressed A549 (Fig 5D) and NCI-H226 cells (Fig 5E). In contrast, the expression level of TRIM21 did not influence the expression of hPCL3L. In addition, we examined whether the hPCL3S and hPCL3L protein stability could be modulated by TRIM21 using a cycloheximide (CHX) chase assay. As showed in Fig 5F, hPCL3S protein sharply decreased within ∼2 h in TRIM21-overexpressed A549 and NCI-H226 cells compared with control cells (Fig 5G and H). Whereas overexpression of TRIM21 does not affect the half-life of hPCL3L in A549 (Fig 5I) and NCI-H226 cells (Fig 5J).

### TRIM21 mediates K48-linked ubiquitination of hPCL3S and K63-linked ubiquitination of hPCL3L

**Figure 6.**
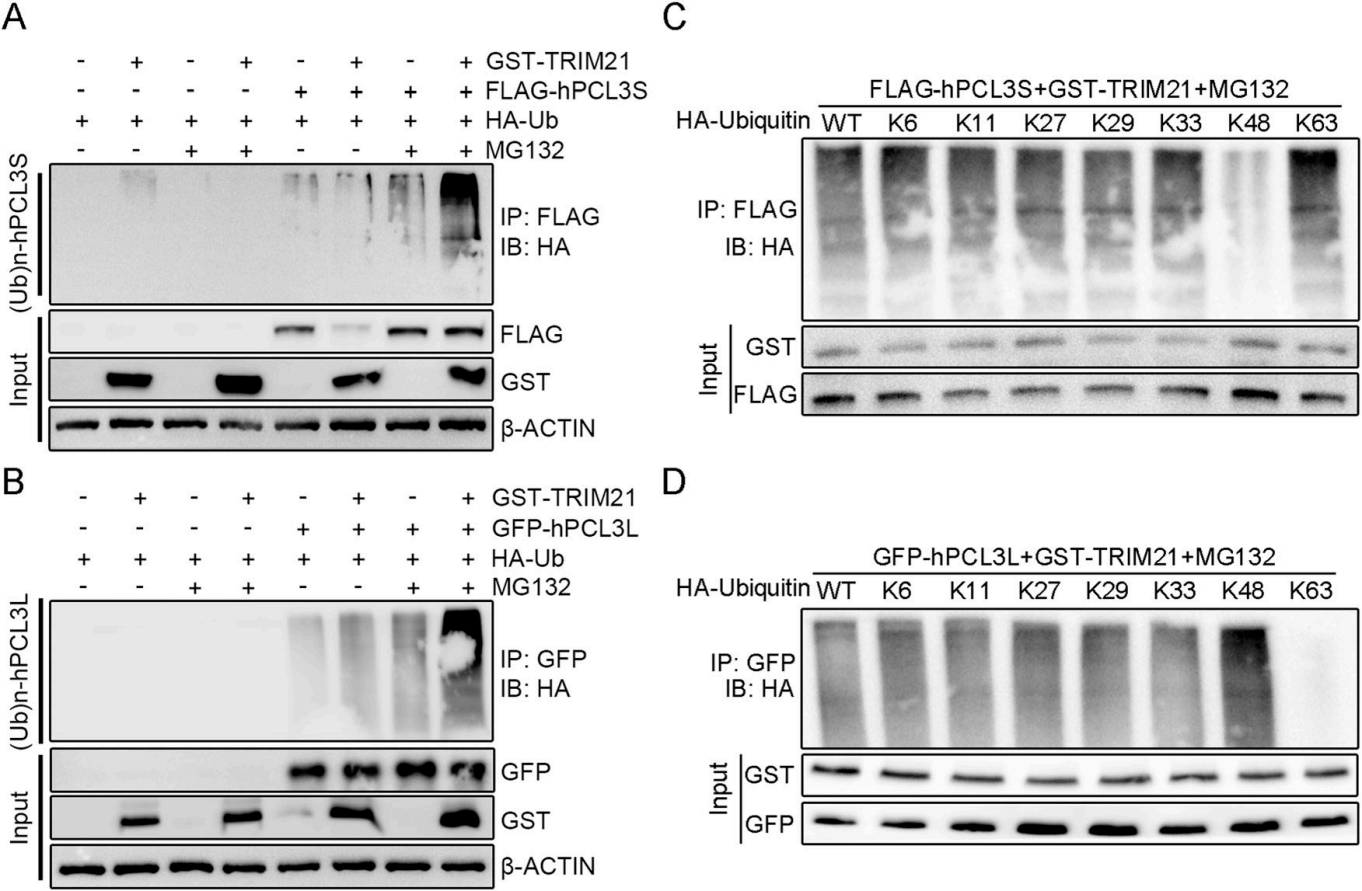
TRIM21 mediates K48-linked ubiquitination of hPCL3S and K63-linked ubiquitination of hPCL3L. **(A)** 293T cells were co-transfected with or without vectors encoding GST-tagged TRIM21, FLAG-tagged hPCL3S, and HA-tagged ubiquitin. Before lysis, cells were treated with or without MG132 for 24 h. Whole cell extracts were subjected to anti-FLAG immunoprecipitation and followed by immunoblotting with anti-HA, GST, and FLAG antibodies. **(B)** 293T cells were co-transfected with or without vectors encoding GST-tagged TRIM21, GFP-tagged hPCL3L, and HA-tagged ubiquitin. Before lysis, cells were treated with or without MG132 for 24 h. Whole cell extracts were subjected to anti-GFP immunoprecipitation. **(C)** hPCL3S ubiquitination reactions with WT, K6, K11, K27, K29, K33, K48, or K63 ubiquitin were precipitated and analyzed by immunoblotting. **(D)** hPCL3L ubiquitination reactions with WT, K6, K11, K27, K29, K33, K48, or K63 ubiquitin were precipitated and analyzed by immunoblotting.

Furthermore, we speculated that TRIM21 might regulate hPCL3S and hPCL3L expression by participating in their ubiquitination. Thus, co-IP and in vitro ubiquitination assay were conducted and revealed that the up-regulation of TRIM21 increased the ubiquitination level of hPCL3S (Fig 6A) and hPCL3L (Fig 6C), whereas TRIM21 overexpression only resulted in the degradation of hPCL3S (Fig 6A), the expression level of hPCL3L was not changed (Fig 6C).

Ubiquitination leads to diverse cellular processes dependent on the linkages in the ubiquitin chain (French et al, 2021). Given that hPCL3S and hPCL3L showed distinguish fate after TRIM21 knockdown or overexpression, we postulated ubiquitin linkages on hPCL3S and hPCL3L are different. There are seven distinct ubiquitin lysine residues associated with ubiquitin linkages (K6, K11, K27, K29, K33, K48, and K63). Thus, we investigated the type of linkage in hPCL3S by transfecting FLAG-hPCL3S and GST-TRIM21 in 293T cells along with ubiquitin mutants containing arginine substitution at the indicated position. Co-IP and Western blotting indicated that the polyubiquitin linkage profiles from K6, K11, K27, K29, K33, and K63 ubiquitin showed consist with WT ubiquitin, but mutations in K48 of ubiquitin severely impaired hPCL3S ubiquitination (Fig 6B). A different result was obtained from hPCL3L. The polyubiquitin linkage profiles from K6, K11, K27, K29, K33, and K48 ubiquitin showed normal performance, nevertheless, mutations in K63 of ubiquitin severely impaired hPCL3L ubiquitination (Fig 6D). These results revealed that hPCL3S is predominantly modified by K48-linked ubiquitin and hPCL3L predominantly modified by K63-linked ubiquitin.

## Discussion

Lung cancer is a cellularly and molecularly heterogeneous disease and is the leading cause of cancer-related death owing to its enduring challenges for diagnosis, increasing rate of recurrence, high incidence of metastasis, and developing resistance to chemotherapy. NSCLC is the most predominant subtype (80%) of lung cancer. The 5-yr survival rate of NSCLC patients remains less than 15%. hPCL3 was an important component of the PRC2 complex. Many studies found that aberrant expression of hPCL3 leads to tumor development, metastasis, and poor prognosis in many cancers. In our previous studies, we also found that silence of hPCL3 suppresses cellular proliferation, migration, and xenograft growth and promotes programmed cell death in ovarian cancer cells (Tao et al, 2018). In this study, we examined the role of two hPCL3 isoforms: hPCL3S and hPCL3L in NSCLC. We found that a high level of *hPCL3S* or *hPCL3L* expression was associated with lower overall survival in NSCLC. Moreover, hPCL3S and hPCL3L were up-regulated in clinical NSCLC samples. Meanwhile, both hPCL3S and hPCL3L showed accelerating colony formation, cell cycle arrest, increasing apoptosis, and migration of NSCLC cells in vitro. Furthermore, in vivo assays indicated that knockout of hPCL3S or hPCL3L cells suppressed the tumorigenicity and migration of NSCLC cells in nude mice. Importantly, the hPCL3S knockdown showed significantly more effective suppressive function than hPCL3L knockdown, suggesting that hPCL3S could be a more powerful potential therapeutic target in NSCLC than hPCL3L.

Subsequently, the subcellular location of hPCL3S and hPCL3L has been studied. Our observation showed hPCL3L is only located in the nucleus, whereas hPCL3S is located in both cytosol and nucleus. These results suggest hPCL3S and hPCL3L may have different functions. One of the limitations to the present study is the exact role of hPCL3S in the cytosol is not clear. In addition, the interaction proteins of hPCL3S and hPCL3L have been screened using FLAG affinity purification and mass spectrometry assays, respectively. We confirmed TRIM21 could interact with both hPCL3S and hPCL3L simultaneously. In the future study, we will determine the core domains of hPCL3S and hPCL3L interacting with TRIM21.

Protein ubiquitination is a crucial mechanism that controls many cellular processes, such as protein degradation, DNA repair, chromatin remodeling, cell-cycle regulation, endocytosis, and kinase signaling pathways (Reyes-Turcu et al, 2009). The ubiquitin-activating enzyme (E1), the ubiquitin-conjugating enzyme (E2), and the ubiquitin ligase enzyme (E3) play important role in protein ubiquitination. TRIM21 is a member of the TRIM family which has E3 ubiquitin ligase activity and plays a crucial role in many cancers progression (Alomari, 2021). TRIM21 consists of five domains: RING finger domain which is responsible for the activity of the E3 ligase, B-box, and coiled-coil domains which mediate the oligomerization of TRIM proteins with other proteins and promote the formation of macromolecular complexes, PRY domains, and SPRY domains. The interaction between TRIM21 and hPCL3S or hPCL3L indicates that TRIM21 is responsible for the ubiquitination of hPCL3S and hPCL3L. Moreover, results of our present study showed that hPCL3S expression level was elevated in TRIM21 knockdown A549 and NCI-H226 cells and was reduced in TRIM21-overexpressed A549 cells and NCI-H226 cells. In contrast, the expression level of TRIM21 did not influence the expression of hPCL3L. This implied that the ubiquitination of hPCL3S is different from hPCL3L. There are seven lysine residues (K6, K11, K27, K29, K33, K48, and K63) in an ubiquitin molecule which corresponds to the seven different possible types of isopeptide linkage (French et al, 2021). The ubiquitin linkage determines the specific fate of the targeting proteins and endocytic trafficking. Hence, current study demonstrates the type of linkage in hPCL3S and hPCL3L ubiquitin conjugates. Results showed the type of hPCL3S ubiquitination by TRIM21 was a K48-linked ubiquitin chain which regulates protein levels by signaling a target protein for degradation by the proteosome. The type of hPCL3L ubiquitination by TRIM21 was a K63-linked ubiquitin chain which was considered to play key roles in protein–protein interaction, protein kinase activation (French et al, 2021), and the processes such as DNA repair (Spence et al, 1995) and signal transduction (Voutsadakis, 2012). These findings were consistent with our results that high level of TRIM21 suppresses the expression of hPCL3S but not hPCL3L. This is the first time to demonstrate that TRIM21 mediates the ubiquitination of hPCL3S. In the future study, we will explore the function of TRIM21-guided K63-linked ubiquitination in hPCL3L.

In summary, our current work showed an important role for hPCL3S and hPCL3L in NSCLC. Compared with hPCL3L, hPCL3S exhibited significantly more effective role in promoting NSCLC cell proliferation and metastasis. We also found that TRIM21 could interact with both hPCL3S and hPCL3L and promoted K48-linked ubiquitination of hPCL3S result in degradation by the proteosome. This study suggested that hPCL3S may be a more effective therapeutic target for NSCLC treatment compared with hPCL3L.

## Materials and Methods

### Clinical specimens

Human NSCLC specimens and the adjacent non-cancerous tissues were collected from the First Affiliated Clinical Medical College of Zhejiang Chinese Medical University with informed consent. The study was approved by Ethics Committee of the First Affiliated Clinical Medical College of Zhejiang Chinese Medical University (ethical approval number 2022-KL-099).

### Cell lines and culture

Two NSCLC cell lines including A549 (adenocarcinoma) and NCI-H226 (squamous cell carcinoma) were obtained from the National Collection of Authenticated Cell Culture of Chinese Academy of Sciences. All cells were cultured according to their instructions in an incubator with 95% humidified air containing 5% CO_2_ at 37°C.

### Generation of the knockout cell line with CRISPR/Cas9

gRNA sequences for CRISPR/Cas9 were designed at CRISPick (https://portals.broadinstitute.org/gppx/crispick/public). The sequence of hPCL3S gRNA is 5′-CTCTCCTGCCTCCTCCAGTG-3′. The sequence of hPCL3L gRNA is 5′-GGCCCGGAGAATGGTACCTG-3′. The sequence of control gRNA is 5′-GGGCGAGGAGCTGTTCACCG-3′. The complementary oligonucleotides for gRNA were annealed and cloned into pLentiCRISPRv2 Neo. These plasmids were then co-transfected with psPax2 and pCMV.VSV.G in a ratio of 10:10:1 into HEK293T cells (ATCC) using Lipofectamine 2000 (Thermo Fisher Scientific) or JetPrime (Polyplus). Lentiviral supernatants were harvested in 48 h. A549 cells and NCI-H226 cells were seeded in six-well plates with 80% confluency. Lentiviral supernatants were added into the cells with 8 μg/mL polybrene. Transduced cells were then selected by neomycin for 3–6 d using a concentration based on killing curves. Surviving cells were subjected to single cloning.

### Colony formation assay

hPCL3S-KO, hPCL3L-KO, and control A549 cells (cells/well) and hPCL3S-KO, hPCL3L-KO, and control NCI-H226 (100 cells/well) were seeded into six-well dishes. The medium was refreshed every 2 d. Plates were incubated for 9 d until colonies had formed. Then, the cells were fixed with 4% paraformaldehyde for 30 min at room temperature and stained with 0.5% crystal violet aqueous (Beyotime) for 20 min. The cell colonies with 50 cells or more were counted. After being photographed, the dye was extracted using 33% acetic acid and quantified using a spectrophotometer at 595 nm. The assay was repeated three times for each cell line.

### Cell cycle analysis

hPCL3S-KO, hPCL3L-KO, and control A549 cells (cells/well) and hPCL3S-KO, hPCL3L-KO, and control NCI-H226 were harvested, washed three times in PBS, and then fixed overnight in 70% alcohol. After being fixed, cells were incubated with RNase digestion (200 μg/mL) at 37°C for 1.30 h, then incubated with PI (10 μg/mL) for 30 min. Cells were analyzed by a BD Accuri C6 flow cytometer (BD Biosiences), and cell cycle phase distribution was analyzed by using FlowJo VX0.7 (FlowJo LLC). The experiments were performed independently in triplicate for each cell line.

### Cell apoptosis analysis

Annexin V-FITC Apoptosis Detection Kit (BD Biosciences) was used to quantify the levels of apoptosis according to the manufacturer’s instructions. Briefly, hPCL3S-KO, hPCL3L-KO, and control cells were harvested, washed three times in PBS, and incubated with Annexin V-FITC and propidium iodide. Flow cytometry data were acquired on BD Accuri C6 flow cytometer and the data were analyzed by using FlowJo VX0.7. The experiments were performed independently in triplicate for each cell line.

### In vitro transwell invasion assay

Transwell cell invasion assays were performed as previously described (Tao et al, 2016). Briefly, 24-well plate inserts (8 μm) were pre-coated with 30 μl Matrigel matrix (diluted at 1:3 with serum-free culture medium) (BD Biosciences) and incubated at 37°C to form a gel. Then, cells were suspended with 250 μl and added into the upper transwell chamber. The lower chamber was filled with 500 μl culture medium with 10% FBS. After incubation at 37°C for 24 h, non-invaded cells on the upper chamber were scraped with a cotton swab. Invaded cells were fixed with 100% methanol and stained with 0.05% crystal violet. Images were taken, and the invaded cells were counted manually. The experiments were performed independently in triplicate for each cell line.

### Western blotting analysis

Total protein of treated cells was lysed by RIPA buffer supplemented with protease and phosphatase inhibitors. Equal amounts of proteins were separated by 10% SDS–PAGE and transferred to polyvinylidene fluoride (PVDF) membranes (Millipore), and 5% non-fat milk was used to block PVDF membranes for 1 h at 37°C and incubated overnight at 4°C with primary antibodies. Then, PVDF membranes were washed three times with PBS. Appropriate HRP-linked secondary antibody were added and incubated for 1 h at room temperature. Finally, ECL (Advansta) was used to visualize the bands of the proteins. Quantification of the immunoblots was performed using ImageJ software (version 1.43u). The antibodies used were anti-hPCL3S (SAB1400484, dilution 1,000; Sigma-Aldrich), anti-hPCL3L (#77271, dilution 1,000; CST), anti-TRIM21 (#92043, dilution 1,000; CST), anti-CCND1 (ab134175, 1,000; Abcam), anti-CDK4 (ab108357, 1,000; Abcam), anti-p21 (ab109520, 1,000; Abcam), anti-BAX (ab32503, 1:10,00; Abcam), anti-BAD (ab32445, 1,000; Abcam), anti-BCL2 (ab182858, 1,000; Abcam), anti-MCL1 (ab28147, 1:1,000; Abcam), anti-β-ACTIN (#ab6276, dilution 1:5,000; Abcam).

### Animal studies

All animal procedures were conducted in accordance with the National Institutes of Health Guidelines for the Care and Use of Laboratory Animals and were approved by the Institutional Animal Care and Use Committee of Zhejiang Chinese Medical University (ethical approval number IACUC-2022050706). For the xenograft experiment, 4–6-wk-old male Balb/c nude mice were subcutaneously injected with 5 × 10^6^ of tumor cells (resuspended in PBS with 50% Matrigel) and to the back of the right lower limb. Six mice were performed in each group. Tumor volumes were measured each other day after palpable tumors appeared. After 24 d of post injection, mice were euthanized and tumors were surgically isolated, weighed, and photographed. For experimental lung metastasis, tumor cells were injected into the 4-wk-old Balb/c nude mice through tail veins. 6 wk later, the mice were euthanized, with the liver tissues dissected, fixed in 10% formalin, examined under the microscope for metastatic foci. The use and care of animals were approved by the Laboratory Animal Center of Zhejiang Chinese Medical University (ethical approval number IACUC-2022050706).

### Co-IP

Cells were transiently transfected with the indicated plasmids using Lipofectamine 2000 reagents. After 48 h of cultivation, the cells were washed and resuspended in lysis buffer. Equal amounts of cleared cell lysates were subjected to immunoprecipitation with GFP or FLAG antibody and protein G-Sepharose beads. The immune complexes and input lysates were detected by immunoblot analysis.

### Immunofluorescence assays

A549 and NCI-H225 cells were seeded onto coverslips and washed with PBS and fixed in 4% paraformaldehyde in PBS for 20 min, permeabilized with 0.1% Triton X-100 for 2 min at room temperature and blocked with 5% normal goat serum. Anti-hPCL3S or anti-hPCL3L antibodies were added and incubated at 4°C overnight. After being washed three times in PBS, secondary antibodies were added and incubated for 1 h at room temperature. Finally, the nucleus was stained with 4′,6-diamidino-2-phenylindole (DAPI) for 2 min. Labeled cells were examined under the fluorescence microscope.

### CHX chase assay

Cells were transiently transfected with TRIM21 plasmid or empty control. After 48 h of cultivation, the medium was refreshed before CHX was added. CHX (10 μg/mL) were added to the culture and cells were harvested at the indicated time points. Total protein was extracted as described previously and resolved by Western blotting with following primary antibodies: anti-hPCL3S (SAB1400484, dilution 1:1,000; Sigma-Aldrich), anti-hPCL3L (#77271, dilution 1:1,000; CST), anti-TRIM21 (#92043, dilution 1:1,000; CST), anti-β-ACTIN (#ab6276, dilution 1:5,000; Abcam).

### Ubiquitination assay

For in vitro ubiquitination assays, 293T cells were transfected with the indicated plasmids using Lipofectamine 2000. After 48 h, cells were treated with 0.25 μM MG132 for 24 h. After treatment, cell lysates were immunoprecipitated (IP) with the labeled antibodies. The eluted proteins were determined by Western blotting.

### Statistical analysis

To compare two groups, the normality of the data is first evaluated with the Shapiro–Wilk test, and variance is assessed using the Levene test. If the data are normally distributed, a *t* test is applied. For samples that do not follow a normal distribution, a Mann–Whitney test is used for equal variances, and a median test is used for unequal variances. When more than two groups are compared, a residual test is performed to study normality, and homoscedasticity is assessed with the Levene test. Depending on the distribution of the data, an ANOVA, Brown–Forsythe, Kruskal–Wallis, or median test is performed. Paired comparisons are further explored using post hoc tests, either Dunnet or Bonferroni. All analyses are two tailed. Error bars represent either the SD. Survival analyses are conducted using the log-rank test. All statistical analyses are performed with GraphPad software, version 8 (*) *P* < 0.05, (**) *P* < 0.01, (***) *P* < 0.001.

## Data Availability

No data were used for the research described in the article.

## Supplementary Material

Reviewer comments
